# Intra-hippocampal administration of ZIP alleviates depressive and anxiety-like responses in an animal model of posttraumatic stress disorder

**DOI:** 10.1186/1744-9081-10-28

**Published:** 2014-09-01

**Authors:** Li-Li Ji, Lei Tong, Bao-Ku Xu, Chang-Hai Fu, Wan Shu, Jun-Bo Peng, Zhen-Yu Wang

**Affiliations:** 1 Department of Anatomy, College of Basic Medical Sciences, China Medical University, Shenyang 110001, People’s Republic of China; 2 College of Clinical Medicine, China Medical University, Shenyang 110001, People’s Republic of China

**Keywords:** Posttraumatic stress disorder, Single prolonged stress, ZIP, PKMζ

## Abstract

**Background:**

Given that impairment of fear extinction has been implicated in the pathogenesis of posttraumatic stress disorder (PTSD), effective pharmacological interventions that facilitate fear extinction may provide alternative strategies to conventional treatment. It is generally accepted that the zeta inhibitory peptide (ZIP), a controversial inhibitor of protein kinase M zeta (PKMζ), could erase certain types of previously established long-term memories. However, it is unclear whether ZIP administration may alleviate PTSD-associated depressive and anxiety-like abnormalities.

**Methods:**

Here we developed a re-stressed single-prolonged stress (SPS) paradigm, a modified prevalent animal model of PTSD, and assayed the expressions of PKMζ in the hippocampus after SPS procedure. Next, Seven days prior to re-stress, ZIP was injected into the hippocampus, and the depressive and anxiety-like behavior was examined by the subsequent forced swim (FS), open-field and elevated plus maze (EPM) test.

**Results:**

Rats given ZIP prior to FS exhibited a reduction of immobility time in FS test, and more open arms (OA) entries and longer OA duration in EPM. They also spent longer time in the center of the open field.

**Conclusions:**

Our results suggested that re-stressed SPS could reproduce behavioral alteration similar to that observed in patients with PTSD, and these behavioral symptoms co-morbid with PTSD could be effectively alleviated by the intro-hippocampal administration of ZIP.

## Background

Posttraumatic stress disorder (PTSD) can develop following exposure to a severe traumatic event or natural disasters [1]. It is a psychopathological response to the traumatic stressor and is characterized by intense memories in which patients re-experience their original traumatic experiences, as well as avoidance of the trauma-related stimuli, and increased arousal [2,3]. However, the precise mechanism of the intricate biological and psychological symptom remains elusive. The neural structures involved in the pathophysiology of PTSD belong to the limbic system, a region important for emotional processing in both humans and animals [4]. The three brain regions within the limbic system most clearly altered in PTSD have been identified as the prefrontal cortex, the amygdala and the hippocampus, among which, the hippocampus plays a key role in explicit memories of traumatic events and in mediating learned responses to contextual cues [5-8]. Indeed, hippocampal reduction has been found in patients with PTSD in a majority of structural neuroimaging studies [9-13].

Single-prolonged stress (SPS), a currently prevalent animal model of PTSD, has been extensively developed and employed in the investigation of PTSD [14-16]. SPS consists of three different stress paradigms: restraint for 2 h, forced swim for 20 min, and ether anesthesia. SPS exposure results in impaired extinction of contextual fear [16], enhanced glucocorticoid negative feedback, and enhanced anxiety-like behavior [17,18], partially resembling the pathophysiological and behavioral abnormalities of PTSD [19].

The persistence of traumatic fear memories in PTSD suggests this disorder might be associated with extinction deficits [20-23], consistent with an interpretation of PTSD as a syndrome of deficient extinction ability [24]. Thus, any intervention facilitating fear extinction or disrupting fear memory may have a therapeutic value on PTSD. Over the past two decades, multiple lines of evidence have indicated that protein kinase M zeta (PKMζ), a brain-specific atypical protein kinase C (PKC) isoform, is required for long term potentiation (LTP) and the maintenance of several forms of memory, including the hippocampus-dependent memory [25-31]. Overexpression of PKMζ could enhance long-term memory, while pharmacologically blocking PKMζ by myristoylated zeta-pseudosubstrate inhibitory peptide (ZIP) could erase previously established long-term memories [26,28,32-35]. Although currently there is still an ongoing debate on the critical issue of PKMζ being a major memory maintenance molecule and ZIP as a specific PKMζ inhibitor [34,36-40], there is a consensus among researchers that ZIP can disrupt certain types of memory [29,30,33,34,37,41]. Thus the mechanism of ZIP on memory disruption is far from clear. Nevertheless, PKMζ remains largely to be the target of ZIP infusions [42]. These inconsistent findings open up a variety of opportunities to gain additional insight into the action of ZIP.

As mentioned above, most research on ZIP has been applied to a certain kind of conditioned learning and memory paradigm to investigate its role on memory disruption. Since SPS has been shown to disrupt the retention stage of fear extinction [20], it is interesting to know whether ZIP may have an impact on SPS procedure, especially the depressive and anxiety-like behavior, representing the core symptom of PTSD-related abnormalities. As a result, the present study used a classic SPS model, but after the last quiescent period, the rats were re-exposed to the forced swim component of SPS, which served as the re-stress component of the paradigm. Next, the expression of PKMζ in the hippocampus of SPS rats was assayed. Finally, ZIP was microinfused into the hippocampus seven days prior to re-stress, and subsequent forced swim, open-field and elevated plus maze test were performed, so as to assess the effect of ZIP on the PTSD-associated symptoms of depressive and anxiety-like behavior.

## Materials and methods

### Animals

Male Sprague-Dawley rats (7 - 8 weeks old) weighing 200 - 250 g were purchased from the Experimental Animal Center of China Medical University (Shenyang, China). Animals were housed singly under a 12:12-h light dark cycle, with food and water freely available. Following an adaptation period of at least 7 days, the experimental procedures were undertaken. All procedures complied with the National Institutes of Health *Guide for the care and use of laboratory animals* and were approved by the Animal Care and Use Committee of China Medical University.

### Experimental groups and the SPS model

**Figure 1:**
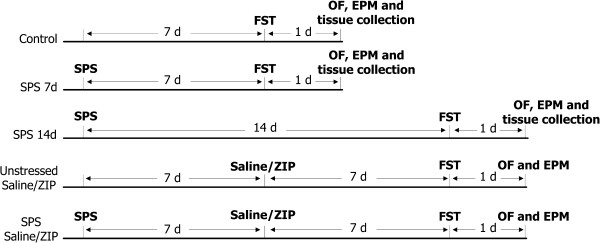
**Schematic of experimental design.** Rats were exposed to control handling or SPS, followed by 7 or 14 days of quiescent period with no manipulation. Next, for the Control, SPS 7d and SPS 14d groups, subsequent forced swim (FS), open-field (OF) and elevated plus maze (EPM) test were performed, and the rats were finally sacrificed for Western blotting and real-time RT-PCR. For the Control + Saline, Control + ZIP, SPS + Saline, and SPS + ZIP groups, ZIP or saline were administrated after the 7 days of quiescent period of SPS. Following another 7 days interval, FS, OF and EPM were performed.

The rats were randomly assigned to seven groups (Control, SPS 7d, SPS 14d, Control + Saline, Control + ZIP, SPS + Saline, and SPS + ZIP, 12 rats per group). The SPS procedure was conducted as described previously [1,17], with slight modifications. Briefly, Rats were restrained for 2 h inside a disposable restraint holder (diameter 58 mm, length 150 mm). Next, they were individually placed in a clear acrylic container (600 × 400 × 500 mm) filled two thirds with water (24°C), and forced to swim for 20 min. Following a 15-min recuperation, animals were exposed to diethyl ether until loss of consciousness and left undisturbed in their cages for 7 or 14 days according to their groups (Figure 1).

### Surgery

Rats were anaesthetized with chloral hydrate (400 mg/kg i.p) and prepared with bilateral stainless steel 26-gauge cannulae aimed at the dorsal hippocampus using stereotaxic coordinates (anteroposterior, -3.6 mm; medial-lateral, ± 3.1 mm; dorsoventral, -2.4 mm) relative to bregma. Cannulae were secured to the skull with stainless steel screws and dental cement. Stainless steel obdurators remained in the cannulae when rats were not being injected to prevent occlusion. Each rat was given a recovery period of at least 7 d before the experiments.

### Drug infusions

ZIP (Abcam, Cambridge, MA, USA) was dissolved in sterile saline to a concentration of 10 nmol/μl. ZIP or saline were infused into the dorsal hippocampus (1 μl per hemisphere) via a microinjector (28 gauge) connected to a microinfusion pump (Stoelting Co., Wood Dale, IL, USA) at a rate of 0.25 μl per min. The injector remained connected for an additional 1 min to allow for drug diffusion away from the tip of the cannula.

### Forced swim test (FST)

Rats were individually forced to swim in an open cylindrical container (diameter 20 cm, height 40 cm) filled to two-thirds with 24°C fresh water. The total duration of immobility during the 5-min test was scored by a trained individual blinded to the experimental group. Each mouse was judged to be immobile when it ceased struggling and remained floating motionless in the water, making only those movements necessary to keep its head above water.

### Open-field test (OFT)

The open-field test was used to quantify locomotor, exploratory and anxiety-like behavior. The apparatus was a black Plexiglas enclosure measuring 50 × 50 × 50 cm with a red fluorescent light illumination over the center of the arena. After 30 min of acclimation in the room, rats were placed in a central start position in the open arena and allowed to explore for 5 min, during which their behavior was recorded and analyzed with “SuperMaze” software (Softmaze Co., Shanghai, China). The arena was cleaned with 70% ethanol after each session and individual rat was tested only once.

### The elevated plus maze (EPM)

The EPM apparatus consisted of two opposing open and two opposing closed arms (50-cm arms, elevated 50 cm off the ground). Animals were placed into the center (10 × 10 cm) of the maze facing an open arm and behavior was recorded for 5 min. The number of arm entries and time spent in open and closed arms were analyzed with “SuperMaze” software (Softmaze Co.). The percentage of time spent in the open arms and percentage of entries into the open arms relative to total (open + closed) arm were quantified as assessments of anxiety.

### Western blot analysis

The rats of each group were decapitated rapidly and the hippocampi were dissected on ice. The samples were homogenized with loading buffer containing 200 mM Tris-buffered saline, 4% sodium dodecyl sulfate, 20% glycerol and 10% 2-mercaptoethanol, and were denatured by boiling for 3 min. The protein fraction (30 μg/lane) extracted from each sample was separated by 12% (w/v) gradient sodium dodecyl sulfate-polyacrylamide gel electrophoresis and transferred to a 0.45-μm polyvinylidene fluoride (PVDF) membrane (Millipore, Billerica, MA, USA). Following blocking with 5% (w/v) skimmed milk in 0.05% TBS with Tween-20 (TBST) at room temperature for 2 h and incubation with a rabbit polyclonal antibody against rabbit polyclonal antibody against PKC (1:5000; Abcam, Cambridge, MA, USA) overnight at 4°C, the membrane was incubated with anti-rabbit IgG-HRP (1:5000; ZSBio, Beijing, China) secondary antibodies for another 2 h at room temperature. Blots were scanned with a ChemiDoc XRS + image analysis system (Bio-Rad Laboratories, Hercules, CA, USA) and analyzed with ImageJ software (National Institutes of Health, Bethesda, MD, USA).

### mRNA extraction and quantitative real-time RT-PCR analysis

For detecting the mRNA levels of PKMζ and PKCζ, quantitative real-time RT*-*PCR was conducted. Total RNA from dissected hippocampus was isolated with TRIzol (Invitrogen, Carlsbad, CA, USA) according to the manufacturer's instructions. The primer sequences (designed and synthesized by Sangon Biotech Co., Shanghai, China) were presented in Table 1. Complementary DNA (cDNA) was obtained from total RNA using PrimeScript™ RT Reagent Kit (TaKaRa Biotech, Dalian, China). Real-time quantitative PCR analysis was performed on the ABI 7500 Real-time PCR System (Applied Biosystems Inc., Foster City, CA, USA), using SYBR^®^*Premix Ex Taq*™ kit (TaKaRa Biotech). Triplicate reactions were run per sample. Relative quantification of gene expression was determined based on the threshold cycle (Ct) value for each PCR reaction. ∆Ct values represent normalized target gene levels with respect to the internal control (GAPDH). Relative quantification of gene expression (relative amount of target RNA) was determined by the 2^-∆Ct^ method. ∆∆Ct values were calculated as the ∆Ct of each sample minus the mean ∆Ct of the calibrator samples (control group). The fold change in expression was calculated with the equation 2^-∆∆Ct^. All primer sets had comparable efficiency of amplification. After amplification, the specificity of the PCR products was verified by a melting curve analysis to ensure that a single product of expected melt curve characteristics was obtained.

**Table 1:** Sense and antisense primers used to amplify each cDNA of interest

	**Sense primer (5’–3’)**	**Antisense primer (5’–3’)**
PKMζ	5’-CCATGCCCAGCAGGACCACC-3’	5’-TGAAGGAAGGTCTACACCATCGTTC-3’
PKCζ	5’-CCTTCTATTAGATGCCTGCTCTCC-3’	5’-TGAAGGAAGGTCTACACCATCGTTC-3’
GAPDH	5’-ACATGGTCTACATGTTCC-3’	5’-CAGATCCACAACGGAATAC-3’

### Statistical analysis

All data were presented as mean ± S.E.M. unless otherwise specified, and were analyzed with SPSS software (version 20.0; IBM, Armonk, NY, USA) and SigmaPlot (version 12.3; Systat Software Inc., San Jose, CA, USA). Data were analyzed via one-way ANOVA or two-way ANOVA, followed by Tukey’s post hoc test or unpaired Student’s *t* test when appropriate. Significant differences between groups were defined by a *p* value less than 0.05.

## Results

### Effect of ZIP on forced swim behavior following the SPS procedure

**Figure 2:**
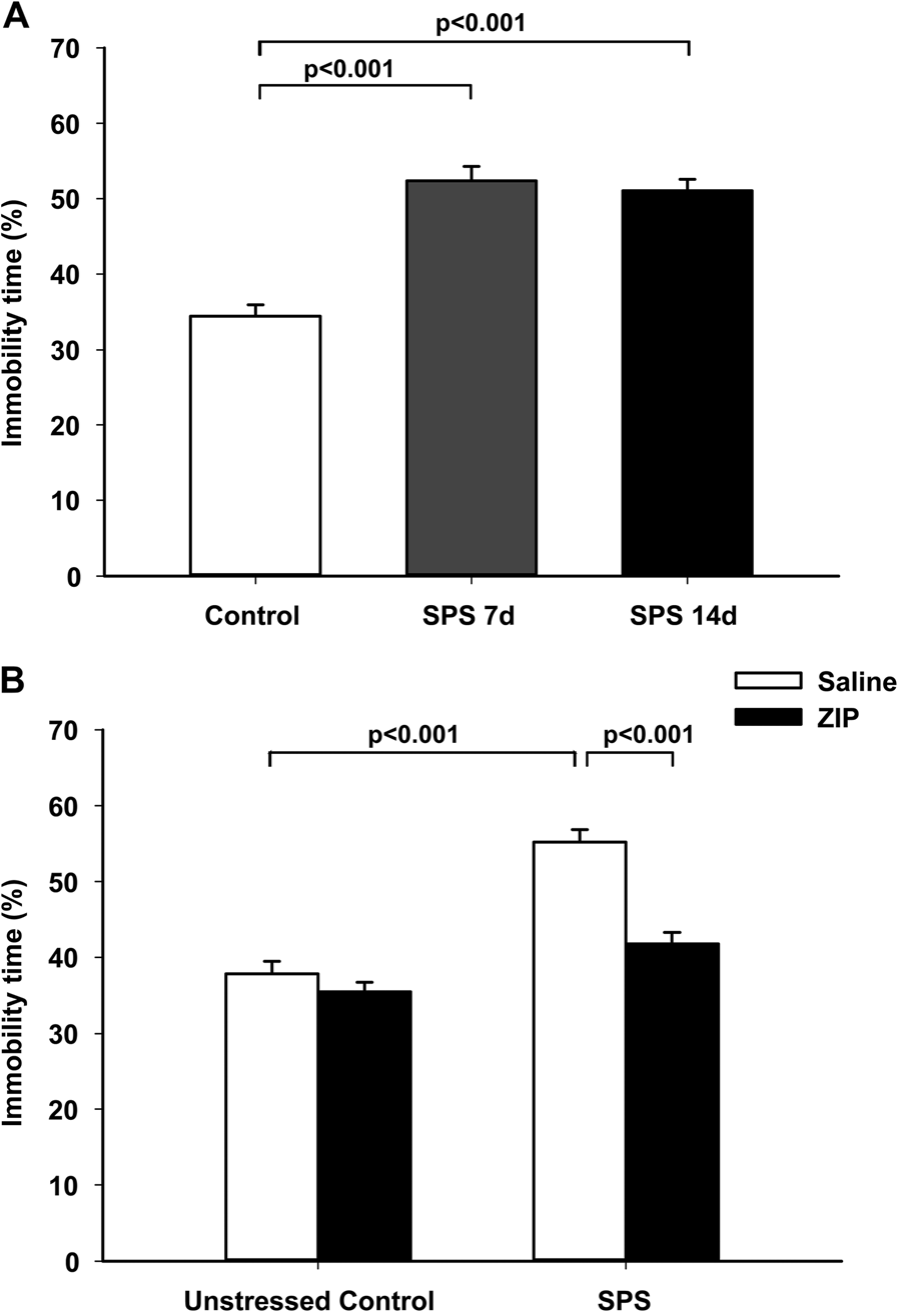
**Comparison of immobility time in the forced swim test.** Data are expressed as mean ± S.E.M. with 12 rats per group. The animals in SPS 7d group or SPS 14d group spent a significantly longer percentage of immobile time (immobile time%) compared with the control group (p < 0.001, respectively) **(A)**. The microinjection of ZIP to the hippocampus resulted in reduced percentage of immobile time in the SPS + ZIP group compared with the ZIP + Saline group (p < 0.001) **(B)**.

To examine the effects of re-exposure to the traumatic stress, seven or fourteen days after SPS stressors were applied, rats were re-stressed by re-exposure to the forced swim component of SPS, and they exhibited enhanced depressive-like behavior evaluated by immobility time in 5-min FST (Figure 2A. Immobility Time (s): Control group 103.3 ± 15.57; SPS 7d group 157.1 ± 20.22; SPS 14d group 153.2 ± 15.85). One-way ANOVA showed significant differences among the groups in immobility time (F (2, 33) = 35.91, *p* < 0.001). Tukey’s post hoc analysis revealed that the animals in SPS 7d group or SPS 14d group spent a significantly longer percentage of time immobile compared with the control group (*p* < 0.001, respectively). There was no significant difference in the immobility time between the two SPS-exposed groups (*p* = 0.849).

Next, the PKMζ inhibitory peptide (ZIP) was microinfused into the bilateral dorsal hippocampi after the seven undisturbed days for SPS and the animals were left undisturbed for another seven days until the FST was carried out (Figure 2B. Immobility Time (s): Control + Saline group 113.4 ± 4.98; Control + ZIP group 106.5 ± 3.55; SPS + Saline group 165.7 ± 4.98; SPS + ZIP group 125.3 ± 4.51). Two-way ANOVA showed significant effects of SPS (F (1, 44) = 61.185, *p* < 0.001), ZIP (F (1, 44) = 27.117, *p* < 0.001), and interaction between SPS and ZIP (F (1, 44) = 13.562, *p* < 0.001) . Tukey’s post hoc analysis revealed that the percentage of immobile time in the SPS + ZIP group was shorter than that in the SPS + Saline group (*p* < 0.001). There was no significant difference in the percentage of immobile time between the SPS + ZIP and Control + Saline groups (*t* (22) = 1.764, *p* = 0.091).

### Effect of ZIP on anxiety-like behavior

**Figure 3:**
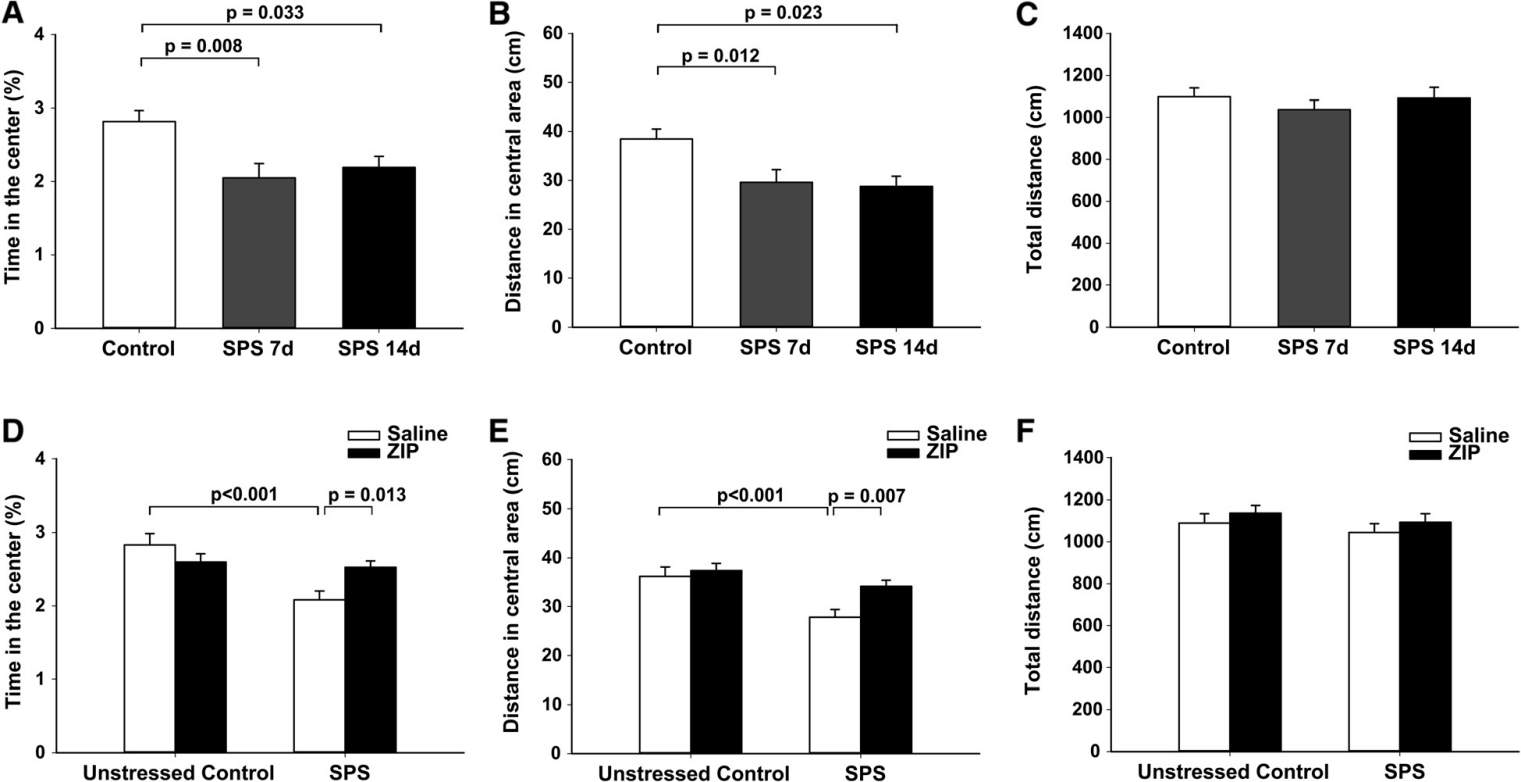
**Effect of ZIP on behavior in the open field test.** Time in the center **(A and D)**, distance in the central area **(B and E)**, and total distance **(C and F)**. Data are expressed as mean ± S.E.M. with 12 rats per group.

Open field test was performed 1 day after FST to examine anxiety-like behavior and locomotion. One-way ANOVA showed significant differences in time in center (F (2, 33) = 5.961, *p* = 0.006) and distance in center (F (2, 33) = 5.776, *p* = 0.007) among the groups (Figure 3A, B. Time in center (s): Control group 8.5 ± 0.45; SPS 7d group 6.2 ± 0.59; SPS 14d group 6.6 ± 0.45. Distance in center (cm): Control group 38.47 ± 2.017; SPS 7d group 29.61 ± 2.582; SPS 14d group 28.76 ± 2.069). Post hoc comparison revealed that both the SPS 7d group and the SPS 14d group spent significantly less time in the center than the control group (*p* = 0.007, p = 0.032, respectively) and covered less distance in the center (*p* = 0.012, *p* = 0.023, respectively). There was no difference between the SPS 7d group and the SPS 14d group (*p* > 0.05). As for the total distance covered, one-way ANOVA did not reveal a significant difference among the three groups (F (2, 33) = 0.505, *p* = 0.608), suggesting that SPS exposure did not affect gross motoric behavior (Figure 3C).

Next we examined whether the ZIP microinjection could alleviate the anxiety-like behavior evaluated by OFT in rats subjected to SPS. Two-way ANOVA showed significant effects of SPS (F (1, 44) = 11.772, *p* < 0.001; F (1, 44) = 13.699, *p* < 0.001), ZIP (F (1, 44) = 0.770, *p* = 0.385; F (1, 44) = 5.743, *p* = 0.021), and interaction between SPS and ZIP (F (1, 44) = 7.877, *p* = 0.007; F (1, 44) = 2.638, *p* = 0.111) in time and distance in the center (Figure 3D, E. Time in center (s): Control + Saline group 8.5 ± 0.46; Control + ZIP group 7.8 ± 0.33; SPS + Saline group 6.2 ± 0.36; SPS + ZIP group 7.6 ± 0.26. Distance in center (cm): Control + Saline group 36.18 ± 1.913; Control + ZIP group 37.38 ± 1.441; SPS + Saline group 27.84 ± 1.591; SPS + ZIP group 34.13 ± 1.236). Tukey’s post hoc analysis revealed that the time in the center in the SPS + ZIP group was higher than that in the SPS + Saline group (*p* = 0.013) , and the distance in the center in the SPS + ZIP group was more than that in the SPS + Saline group (*p* = 0.007), indicative of lower anxiety. There were no significant differences in the total distance (SPS: F (1, 44) = 1.154, *p* = 0.289; ZIP: F (1, 44) = 1.365, *p* = 0.249; interaction between SPS and ZIP: F (1, 44) = 0.0001, *p* = 0.992) (Figure 3F).

**Figure 4:**
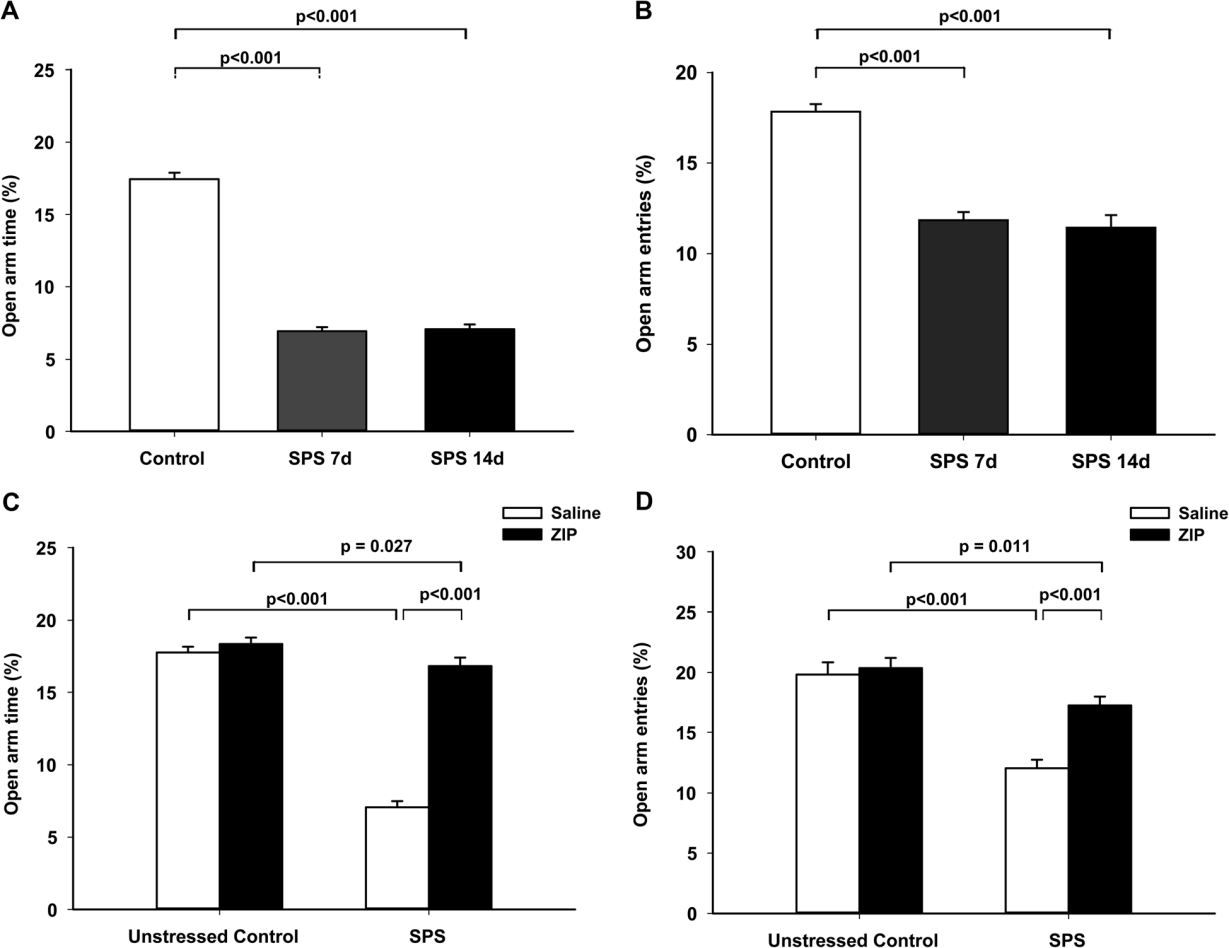
**Comparisons of the percentage of time spent on the open arms (OA time %) (A and C) and the percentage of open arms entries (OA entries %) (B and D) in the elevated plus maze test.** Data are expressed as mean ± S.E.M. with 12 rats per group.

One hour after OFT, the elevated plus maze test was applied. One-way ANOVA showed significant differences in the percentage of open arm time (F (2, 33) = 277.775, *p* < 0.001) and the percentage of open arm entries (F (2, 33) = 44.922, *p* < 0.001) among the groups (Figure 4A, B. Open Arm Time (s): Control group 52.3 ± 1.33; SPS 7d group 20.7 ± 0.91; SPS 14d group 21.2 ± 0.97. Open Arm Entries: Control group 1.25 ± 0.18; SPS 7d group 0.67 ± 0.14; SPS 14d group 0.83 ± 0.17). Post hoc comparison revealed that exposure of rats to SPS led to a reduction in OA duration and OA entries in both the SPS 7d (*p* < 0.001, respectively) and SPS 14d groups (*p* < 0.001, respectively) compared with the unstressed control, indicating an increased anxiety behavior.

After ZIP injection, two-way ANOVA showed significant effects of SPS (F (1, 44) = 166.766, *p* < 0.001; F (1, 44) = 42.666, *p* < 0.001), ZIP (F (1, 44) = 119.016, *p* < 0.001; F (1, 44) = 11.926, *p* = 0.001), and interaction between SPS and ZIP (F (1, 44) = 93.678, *p* < 0.001; F (1, 44) = 7.805, *p* = 0.008) on the percentage of time spent in the open arms and the percentage of open arm entries (Figure 4C, D. Open Arm Time (s): Control + Saline group 53.3 ± 1.21; Control + ZIP group 55.0 ± 1.37; SPS + Saline group 21.2 ± 1.28; SPS + ZIP group 50.4 ± 1.76. Open Arm Entries: Control + Saline group 1.58 ± 0.31; Control + ZIP group 1.67 ± 0.26; SPS + Saline group 0.83 ± 0.17; SPS + ZIP group 1.25 ± 0.22). Post hoc analysis revealed that ZIP treatment significantly increased the percentage of open arm time and the percentage of open arm entries in the SPS + ZIP group compared with the SPS + Saline group (*p* < 0.001, respectively).

### Changes in the expression of PKMζ and PKCζ

**Figure 5:**
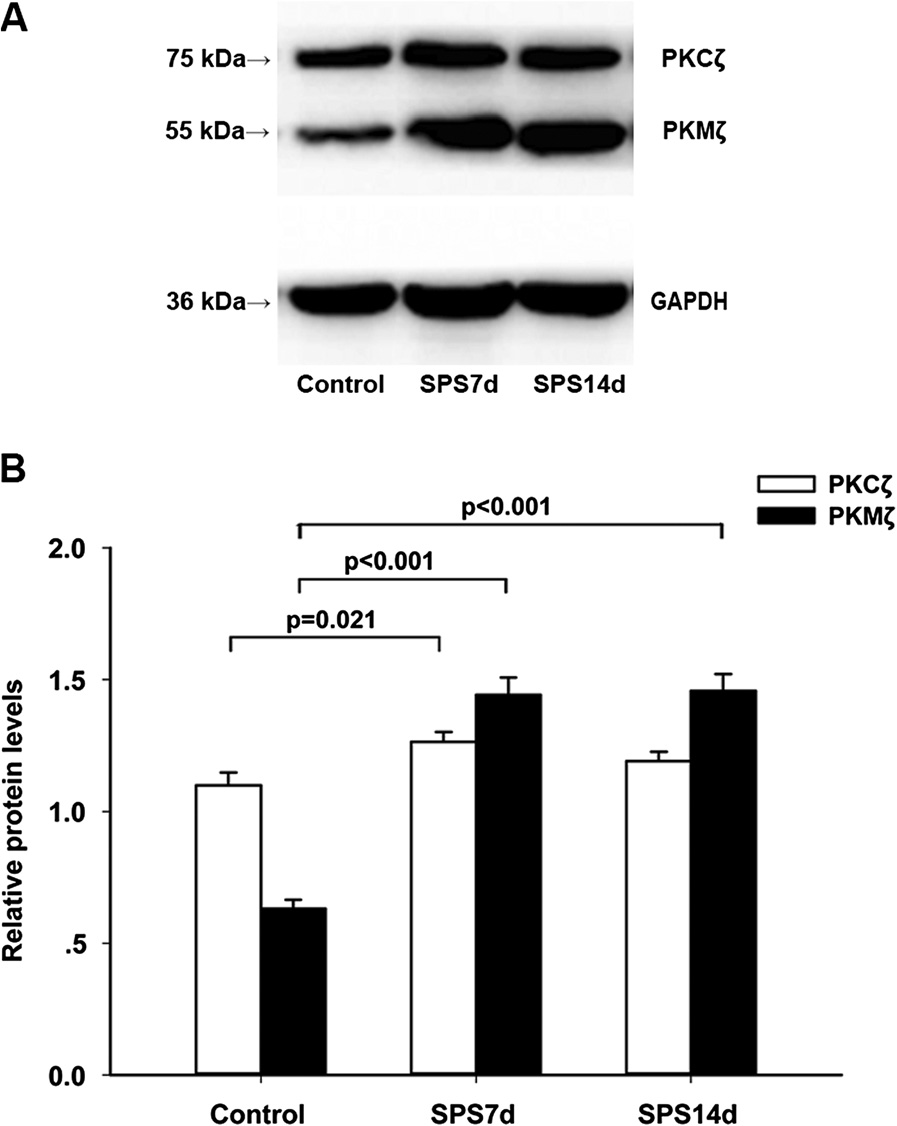
**Western blot analysis demonstrating the expression of PKMζ and PKCζ proteins in the hippocampus of the control, SPS 7d and SPS 14d groups.** GAPDH was used as a loading control **(A)**. The relative levels are presented as the mean ± S.E.M. **(B)**, n = 6 per group.

The PKCζ, PKMζ and GAPDH proteins were detected at 75, 55 and 36 kDa, respectively (Figure 5A), and the mean values of the band densities of the control group were set as 100%. The data were expressed as normalized ODs (Figure 5B). One-way ANOVA showed significant differences in the OD value of PKCζ (F (2, 33) = 4.05, *p* = 0.027). Post hoc comparison revealed that the OD value of the PKCζ bands had a significant increase at 7 day in the SPS groups compared with the control group (*p* = 0.021), while there was no difference between SPS 14d and the control groups (*p* = 0.263). The OD value of the PKMζ bands had a significant increase at 7 and 14 days in the SPS groups compared with the control group (*p* < 0.05, one-way ANOVA). There were also significant differences in the OD value of PKMζ (F (2, 33) = 68.018, *p* < 0.001). Post hoc comparison revealed that both the SPS 7d group and the SPS 14d group had a significant increase in the OD value than the control group (*p* < 0.001, respectively).

**Figure 6:**
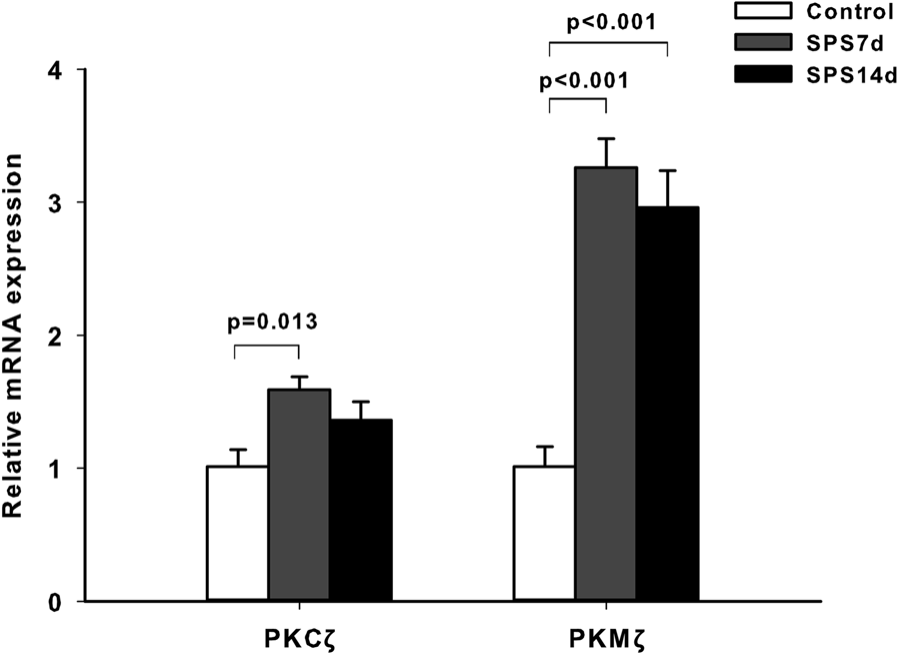
**Effect of SPS on the PKMζ and PKCζ mRNA expression in the hippocampus.** Data are expressed as mean ± S.E.M. with 6 rats per group. Results were compared with GAPDH as the internal control. The relative mRNA levels were represented as the ratios by comparing the expression of each group with that of the Control group.

To further confirm the changes in PKMζ expression caused by SPS exposure, Real-time RT-PCR analysis was performed (Figure 6). One-way ANOVA showed significant differences in the mRNA expression of PKMζ (F (2, 15) = 29.99, *p* < 0.001). Post hoc comparison revealed that both the SPS 7d group and the SPS 14d group had a higher mRNA level than the control group (*p* < 0.001, respectively). In the analysis of PKCζ, one-way ANOVA showed significant differences in the mRNA expression of PKCζ (F (2, 15) = 5.503, *p* = 0.016). Post hoc comparison revealed that the SPS 7d group had a higher mRNA level than the control group (*p* = 0.013), while there was no difference between SPS 14d and the control groups (*p* = 0.15).

## Discussion

In this study, rats subjected to SPS showed significantly enhanced depressive or anxiety-like behavior when re-stressed by re-exposure to the forced swim component of SPS. Meanwhile, there were significantly increased PKMζ levels compared with the control group. The additional FST at the end of SPS quiescent period differed from the conventionally used SPS protocol. After SPS, rats treated with ZIP prior to the trauma-related stressor displayed significantly shorter immobility time in the forced swim test compared with the vehicle group. The inactivation of PKMζ with intra-hippocampal ZIP infusions effectively reduced PTSD-related behavioral abnormalities.

Although recent research in PTSD has yielded a number of important data, many issues concerning the etiopathogenisis remain elusive. In this context, the establishment of an appropriate animal model of PTSD is a prerequisite for better understanding of its mechanisms and the exploitation of more effective therapeutic intervention. Early animal models, like inescapable shock-learned helplessness models, though having good face validity, failed to reproduce the HPA axis changes characteristic of PTSD [17]. Although no ideal animal model of PTSD has been established to date, the single prolonged stress paradigm, proposed by Liberzon et al., has been widely accepted as a putative model for PTSD. SPS model successfully replicates several memory impairments found in PTSD patients, as well as pathophysiological characteristics, such as hypersensitive glucocorticoid feedback. Furthermore, SPS model has been demonstrated to replicate the inner neuroendocrine abnormality associated with PTSD [43]. However, studies using classical SPS protocol alone suggested that SPS might not increase anxiety-related behavior [44-48]; neither could it fully mimic the behavioral alterations without re-exposure to the context that PTSD patients frequently encountered. Antelman et.al. reported that stress-re-stress or a time dependent sensitization (TDS) model was a better paradigm for PTSD [49]. In this model, a single exposure to stress caused subsequent sensitization during the re-stress. Similarly, Harvey et al. used a modified SPS-re-stress paradigm to exhibit enhanced anxiety-related behavior by re-stressing rats with re-exposure to the forced swim component of SPS [44]. Thus, re-exposure to the FST procedure may serve as a trauma-related cue that enhances anxiety-related reactivity [50]. In the present study, we employed the modified SPS protocol. Our data showed enhanced depressive or anxiety-like behavior by re-exposure to the trauma-related stress, consistent with previous reports [15,17,18,44,46,50]. In addition, Wang et al. adopted a single inescapable electric foot shock at the end of SPS stressors, and observed an increase in anxiety-related behavior in the EPM [46,47]. Indeed, varient re-stress after SPS exposure has been demonstrated to be sufficient to produce long-lasting enhanced anxiety-like behavior [50,51].

Multiple lines of evidence indicate that PTSD is associated with memory impairment, as well as reduced hippocampal volumes and abnormal hippocampal function [5]. The hippocampus is responsible for explicit memory processes and context encoding during fear conditioning [6,52,53]. Importantly, the hippocampus seems to interact with the amygdala during the encoding of emotional memories [54-56], a process that is highly relevant to the study of PTSD. Hippocampus-related memory modulation in animals can result from intensive stressors or increased stress-associated hormones [5]. Extensive research has shown that stress is a potent modulator of learning and memory processes–including impairing and facilitating effects [57], which is possibly involved in the development of PTSD.

In the past few years, protein kinase M zeta, which is expressed exclusively in neural tissue and enriched in the forebrain, has attracted intense interest for its putative role in memory maintenance. It lacks pseudosubstrate-dependent inhibition [58,59]. This potential autonomous activity suggests that PKMζ might be an important player in LTP maintenance. Indeed, its activation enhances and its inhibition disrupts previously stored memories [60]. A wealth of subsequent work has indicated that inhibition of PKMζ disrupts long-term memory in a wide range of brain organs, including the hippocampus, amygdala, and insular cortex [60]. The most commonly used inhibitor of PKMζ is myristoylated zeta-pseudosubstrate inhibitory peptide, a synthetic peptide that mimics the pseudosubstrate sequence of PKCζ [25,61], whereas recent work has raised concerns regarding both the role of PKMζ in memory maintenance and the specificity of the pharmacological agent used to inhibit PKMζ in those studies [37,62]. These results, however, do not convincingly exclude the possibility that PKMζ is a key player in memory maintenance [63], and PKMζ still remains largely to be the target of ZIP infusions [42]. But unfortunately, previous studies commonly used a scrambled version of ZIP (Scr-ZIP) as a control peptide, which was demonstrated to be equally effective at reversing LTP as ZIP, or at least, weaken the memory to a certain degree [37]. On the contrary, the saline condition may be a more appropriate control for assessing the effects of PKMζ inhibition, as the animals in saline control routinely exhibited normal retention of conditioned fear [64].

As a result, the present study used saline injection as the control treatment, so as to avoid the vague role of Scr-ZIP. We showed that PKMζ levels were significantly increased one day after re-exposure to the trauma-related stressor. Inactivation of PKMζ with intra-hippocampal ZIP infusions effectively reduced PTSD-related behavioral abnormalities 7 days later, consistent with previous studies that have established the hippocampus as a key structure in context fear memory storage [65-70]. Alternatively, a number of contrary studies implied that the dorsal hippocampus was not an essential component in context fear storage [64]. In spite of the current debate, it is important to point out that the present study places emphasis on the effect of ZIP on the PTSD-like behavior by SPS exposure, rather than the erasure of existing memory. To the best of our knowledge, little literature is concerned with the effect of ZIP on PTSD symptom. Recently, Cohen et al. injected ZIP into four different brain structures after predator scent stress exposure, namely the dorsal hippocampus (DH), basolateral amygdala, lateral ventricle (LV) and insular cortex (IC) [71]. They reported that inactivation of PKMζ in the LV or DH within 1 h of exposure effectively reduced PTSD-like behavioral disruption and trauma cue response 8 days later, while inactivation of PKMζ 10 days after exposure had equivalent effects only when administered in the IC [71]. However, our results were not quite consistent with this comprehensive research. The discrepancy might due first to the different PTSD models applied. The predator scent stress exposure (PSS) model employed by Cohen et al. was comparatively different from the present re-stressed SPS model, which involved different neural circuit and might yield negative impact on the effectiveness of ZIP. Another possibility existed in the adoption of different control groups. As mentioned above, Volk et al. has demonstrated that Scr-ZIP plays a role in the memory disruption to a certain degree [37]. Therefore, Scr-ZIP might not be the most appropriate control for ZIP from the current point of view, which would consequently be hard to make a peer to peer comparison.

In conclusion, the results of the present study demonstrate that intro-hippocampal administration of ZIP after re-stressed SPS effectively alleviates depressive and anxiety-like behavioral abnormalities, and PKMζ might be involved in the development of PTSD. Continued evidence for the controversial mechanism of ZIP will highlight its potential use in a wide range of psychiatric disorders.

## Competing interest

The authors declare that they have no competing interest.

## Authors’ contributions

L-LJ and Z-YW designed the study, wrote the protocol and supervised its execution. B-KX and C-HF worked rat SPS model and isolated the samples. WS and J-BP performed the surgery and behavior test. LT undertook statistical analyses and constructed the figures and table; L-LJ composed the first draft of this manuscript. All authors contributed to and have approved the final manuscript.
